# Supplementary material for: Rapid identification and methicillin resistance test to Staphylococcus aureus in cerebrospinal fluid by MALDI-TOF MS
Source: Microbiol Spectr. 2026 Apr 3;14(5):e01508-25. doi: 10.1128/spectrum.01508-25 (PMC13141823; doi:10.1128/spectrum.01508-25)
Supplement: Supplemental material — Fig. S1 and S2; Tables S1 to S11. [file spectrum.01508-25-s0001.docx]

Figure S1. Enrichment efficiency of centrifugation.


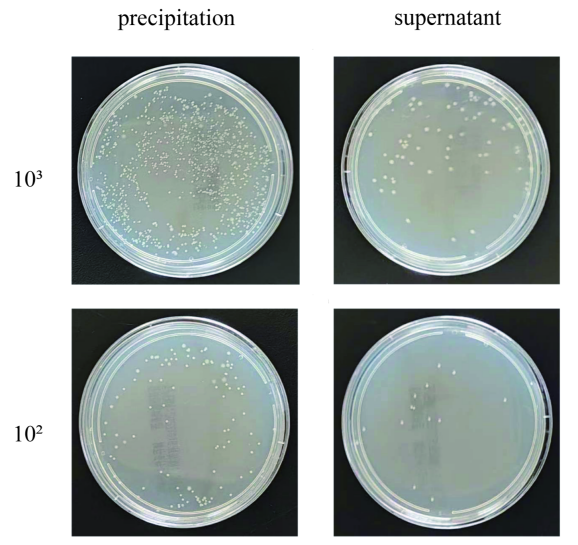

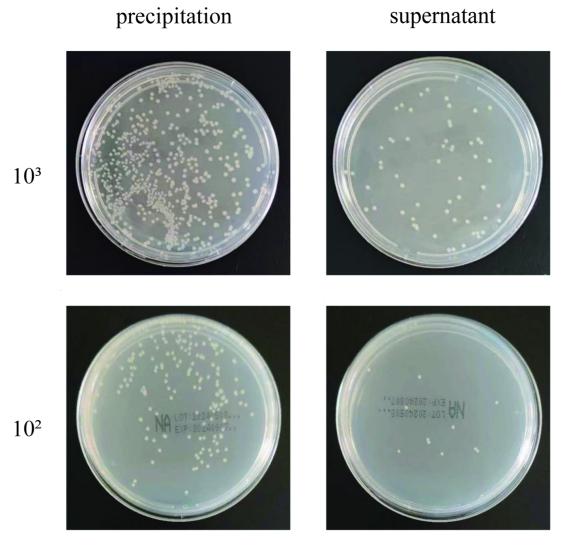


Photographs of culture plates of the enriched pellets and supernatant for S. aureus, within varying concentrations. When the concentration is 10^3^ CFU/mL, the respective enrichment efficiencies are 86.2% and 88.7%. Similarly, when the concentration is 10^2^ CFU/mL, the respective enrichment efficiencies are 89.1% and 92.4%.

Figure S2. Diagnostic workflow for identification and drug resistance analysis of S. aureus in a true diagnostic setting.


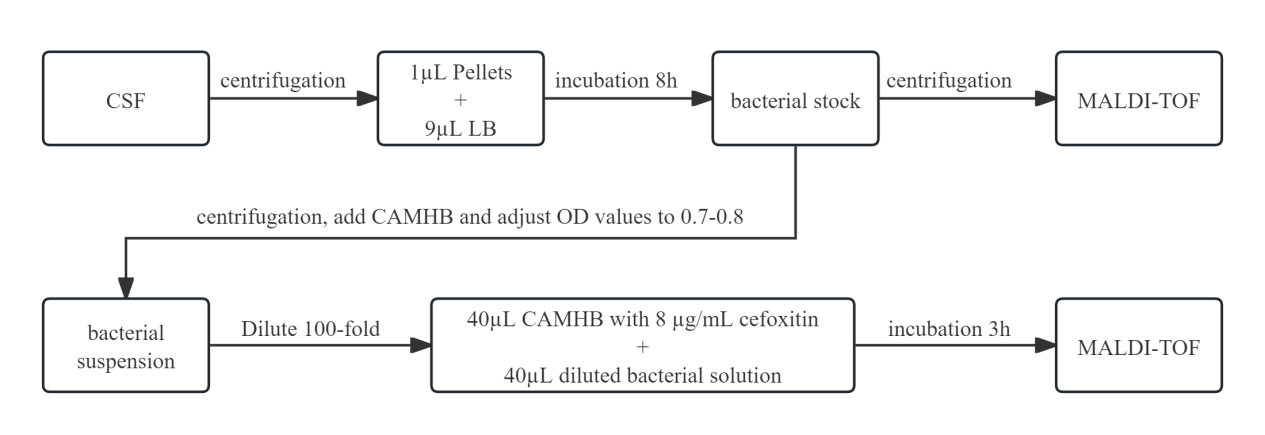


Table S1. OD_600_ values of pathogens in different volumes of LB broth and cerebrospinal fluid

|  | 5h | | 6h | | 7h | | 8h | |
| --- | --- | --- | --- | --- | --- | --- | --- | --- |
| 9µL LB | 0.14 | 0.11±0.03 | 0.38 | 0.29±0.08 | 1.10 | 1.12±0.10 | 2.24 | 2.53±0.33 |
|  | 0.11 |  | 0.22 |  | 1.03 |  | 2.89 |  |
|  | 0.08 |  | 0.27 |  | 1.22 |  | 2.46 |  |
| 19µL LB | 0.09 | 0.07±0.03 | 0.26 | 0.21±0.45 | 0.87 | 0.89±0.18 | 2.34 | 2.14±0.22 |
|  | 0.04 |  | 0.21 |  | 1.08 |  | 2.17 |  |
|  | 0.08 |  | 0.17 |  | 0.72 |  | 1.91 |  |
| 39µL LB | 0.04 | 0.04±0.02 | 0.11 | 0.19±0.09 | 0.64 | 0.66±0.14 | 2.25 | 2.05±0.19 |
|  | 0.06 |  | 0.19 |  | 0.81 |  | 1.88 |  |
|  | 0.03 |  | 0.28 |  | 0.53 |  | 2.02 |  |
| 9µL CSF | 0.00 | 0.01±0.01 | 0.02 | 0.01±0.01 | 0.00 | 0.01±0.01 | 0.01 | 0.01±0.01 |
|  | 0.01 |  | 0.01 |  | 0.02 |  | 0.00 |  |
|  | 0.01 |  | 0.00 |  | 0.01 |  | 0.01 |  |
| 19µL CSF | 0.00 | 0.01±0.01 | 0.00 | 0.01±0.01 | 0.02 | 0.01±0.01 | 0.01 | 0.01±0.01 |
|  | 0.02 |  | 0.01 |  | 0.00 |  | 0.01 |  |
|  | 0.01 |  | 0.02 |  | 0.01 |  | 0.00 |  |
| 39µL CSF | 0.00 | 0.01±0.01 | 0.01 | 0.01±0.01 | 0.02 | 0.01±0.01 | 0.01 | 0.01±0.01 |
|  | 0.01 |  | 0.02 |  | 0.01 |  | 0.00 |  |
|  | 0.01 |  | 0.00 |  | 0.00 |  | 0.01 |  |

The 1 microliter of specimen added to the LB or CSF diluent is the original CSF specimen with S. aureus incorporated in the specimen. the CSF diluent is a confirmed bacterial culture-negative CSF specimen.

Table S2. MIC value distribution of cefoxitin in experimental strains.

| antibiotic | MIC value  (µg/mL) | S. aureus | |
| --- | --- | --- | --- |
|  |  | MSSA (20) | MRSA (20) |
| cefoxitin | MIC range | 2-4 | 8-＞256 |
|  | MIC50 | 2 | 16 |
|  | MIC90 | 4 | 128 |

Table S3. The MALDI-TOF MS analysis results of the short-term bacterial culture in CAMHB^a^.

| Species  (amt of CHAMB [µL]) | Results by incubation time |  |  |  |  |  |
| --- | --- | --- | --- | --- | --- | --- |
|  | 2h |  |  | 3h |  |  |
|  | OD_600_ values | No. of spots  identified^b^ | % | OD_600_ values | No. of spots  identified | % |
| S. aureus (10) | 0.08±0.02  0.07±0.03  0.11±0.02 | 0  1  2 | 20 | 0.44±0.06  0.32±0.04  0.27±0.07 | 3  3  5 | 73.3 |
| S. aureus (20) | 0.06±0.02  0.09±0.03  0.07±0.03 | 3  0  2 | 33.3 | 0.29±0.09  0.16±0.07  0.33±0.03 | 5  3  5 | 86.7 |
| S. aureus (40) | 0.06±0.01  0.05±0.03  0.08±0.02 | 4  3  3 | 66.7 | 0.22±0.06  0.34±0.08  0.19±0.04 | 5  5  5 | 100 |
| S. aureus (80) | 0.04±0.02  0.06±0.01  0.04±0.03 | 2  3  2 | 46.7 | 0.17±0.05  0.29±0.07  0.15±0.05 | 5  5  4 | 93.3 |
| S. aureus (160) | 0.06±0.02  0.04±0.03  0.06±0.03 | 3  4  3 | 66.7 | 0.18±0.05  0.31±0.09  0.28±0.04 | 5  5  5 | 100 |

^a^Five parallel target spots were analyzed.

^b^Out of 5 target spots.

Table S4. OD_600_ value of MRSA and MSSA in CAMHB.

|  | MSSA | MSSA + cefoxitin | *P*-value | MRSA | MRSA + cefoxitin | *P*-value |
| --- | --- | --- | --- | --- | --- | --- |
| 1h | 0.01 | 0.02 | 0.643 | 0.01 | 0.02 | 0.725 |
|  | 0.03 | 0.02 |  | 0.01 | 0.01 |  |
|  | 0.02 | 0.03 |  | 0.03 | 0.03 |  |
| 2h | 0.09 | 0.03 | 0.001 | 0.11 | 0.06 | 0.051 |
|  | 0.12 | 0.02 |  | 0.07 | 0.05 |  |
|  | 0.10 | 0.02 |  | 0.08 | 0.07 |  |
| 3h | 0.32 | 0.02 | 0.001 | 0.25 | 0.21 | 0.219 |
|  | 0.41 | 0.01 |  | 0.27 | 0.19 |  |
|  | 0.32 | 0.02 |  | 0.41 | 0.28 |  |
| 4h | 0.63 | 0.03 | 0.001 | 0.54 | 0.38 | 0.245 |
|  | 0.74 | 0.02 |  | 0.56 | 0.61 |  |
|  | 0.81 | 0.02 |  | 0.94 | 0.45 |  |
| 5h | 1.12 | 0.02 | 0.001 | 0.98 | 0.71 | 0.086 |
|  | 1.01 | 0.04 |  | 0.87 | 0.65 |  |
|  | 1.17 | 0.02 |  | 1.23 | 0.87 |  |
| 6h | 1.72 | 0.02 | 0.001 | 1.96 | 1.28 | 0.112 |
|  | 1.93 | 0.01 |  | 1.41 | 1.04 |  |
|  | 1.71 | 0.03 |  | 1.32 | 1.07 |  |
| 7h | 2.27 | 0.02 | 0.001 | 2.78 | 2.02 | 0.024 |
|  | 2.68 | 0.01 |  | 2.33 | 1.66 |  |
|  | 2.32 | 0.05 |  | 2.61 | 2.08 |  |
| 8h | 3.77 | 0.05 | 0.001 | 3.82 | 2.64 | 0.007 |
|  | 3.96 | 0.03 |  | 3.36 | 2.17 |  |
|  | 3.91 | 0.02 |  | 3.87 | 2.71 |  |

Table S5. OD_600_ values of 20 strains MRSA.

| Number | MIC  (µg/mL) | CAMHB | | | CAMHB (4 mg/L cefoxitin) | | |
| --- | --- | --- | --- | --- | --- | --- | --- |
|  |  | 2h | 3h | 4h | 2h | 3h | 4h |
| R-1 | 8 | 0.08 | 0.19 | 0.62 | 0.05 | 0.13 | 0.19 |
| R-2 | 32 | 0.07 | 0.31 | 0.78 | 0.07 | 0.23 | 0.48 |
| R-3 | 16 | 0.06 | 0.29 | 0.65 | 0.06 | 0.16 | 0.23 |
| R-4 | 8 | 0.13 | 0.41 | 0.71 | 0.03 | 0.11 | 0.17 |
| R-5 | 128 | 0.08 | 0.35 | 0.78 | 0.10 | 0.33 | 0.66 |
| R-6 | 64 | 0.06 | 0.37 | 0.87 | 0.08 | 0.34 | 0.51 |
| R-7 | 64 | 0.04 | 0.13 | 0.55 | 0.06 | 0.28 | 0.49 |
| R-8 | 32 | 0.11 | 0.39 | 0.76 | 0.10 | 0.33 | 0.53 |
| R-9 | 8 | 0.04 | 0.16 | 0.66 | 0.04 | 0.18 | 0.26 |
| R-10 | 32 | 0.07 | 0.32 | 0.73 | 0.07 | 0.27 | 0.55 |
| R-11 | 16 | 0.14 | 0.43 | 0.91 | 0.08 | 0.25 | 0.33 |
| R-12 | 16 | 0.10 | 0.41 | 0.88 | 0.06 | 0.21 | 0.44 |
| R-13 | 32 | 0.09 | 0.40 | 0.68 | 0.08 | 0.26 | 0.49 |
| R-14 | 16 | 0.07 | 0.32 | 0.74 | 0.03 | 0.20 | 0.28 |
| R-15 | 128 | 0.12 | 0.46 | 0.78 | 0.13 | 0.36 | 0.70 |
| R-16 | 256 | 0.06 | 0.36 | 0.87 | 0.08 | 0.42 | 0.72 |
| R-17 | 8 | 0.05 | 0.27 | 0.63 | 0.04 | 0.12 | 0.16 |
| R-18 | 64 | 0.12 | 0.43 | 0.86 | 0.06 | 0.28 | 0.51 |
| R-19 | 8 | 0.08 | 0.30 | 0.71 | 0.03 | 0.13 | 0.21 |
| R-20 | 16 | 0.06 | 0.29 | 0.74 | 0.05 | 0.22 | 0.30 |
|  |  | 0.08±0.03 | 0.34±0.09 | 0.76±0.10 | 0.07±0.03 | 0.24±0.09 | 0.41±0.18 |

CAMHB VS CAMHB (4 mg/L cefoxitin): 2h (*P*=0.700), 3h (*P*=0.003), 4h (*P*=0.001).

Table S6. OD_600_ values of 20 strains MSSA.

| Number | MIC  (µg/mL) | CAMHB | | | CAMHB (4 mg/L cefoxitin) | | |
| --- | --- | --- | --- | --- | --- | --- | --- |
|  |  | 2h | 3h | 4h | 2h | 3h | 4h |
| S-1 | 2 | 0.13 | 0.41 | 0.82 | 0.03 | 0.02 | 0.03 |
| S-2 | 4 | 0.07 | 0.28 | 0.68 | 0.04 | 0.04 | 0.02 |
| S-3 | 2 | 0.10 | 0.42 | 0.87 | 0.01 | 0.02 | 0.01 |
| S-4 | 2 | 0.08 | 0.34 | 0.78 | 0.02 | 0.03 | 0.03 |
| S-5 | 2 | 0.06 | 0.31 | 0.67 | 0.03 | 0.02 | 0.02 |
| S-6 | 2 | 0.04 | 0.21 | 0.52 | 0.02 | 0.03 | 0.01 |
| S-7 | 4 | 0.05 | 0.27 | 0.55 | 0.01 | 0.02 | 0.04 |
| S-8 | 4 | 0.09 | 0.35 | 0.81 | 0.02 | 0.02 | 0.02 |
| S-9 | 2 | 0.07 | 0.31 | 0.82 | 0.03 | 0.02 | 0.03 |
| S-10 | 4 | 0.05 | 0.25 | 0.66 | 0.02 | 0.01 | 0.00 |
| S-11 | 2 | 0.08 | 0.26 | 0.67 | 0.01 | 0.00 | 0.03 |
| S-12 | 4 | 0.10 | 0.31 | 0.79 | 0.03 | 0.01 | 0.02 |
| S-13 | 2 | 0.06 | 0.25 | 0.64 | 0.02 | 0.02 | 0.03 |
| S-14 | 4 | 0.04 | 0.17 | 0.43 | 0.01 | 0.01 | 0.04 |
| S-15 | 4 | 0.06 | 0.24 | 0.74 | 0.02 | 0.02 | 0.02 |
| S-16 | 4 | 0.08 | 0.26 | 0.55 | 0.00 | 0.01 | 0.02 |
| S-17 | 4 | 0.06 | 0.29 | 0.68 | 0.02 | 0.00 | 0.02 |
| S-18 | 2 | 0.08 | 0.23 | 0.66 | 0.01 | 0.02 | 0.03 |
| S-19 | 2 | 0.10 | 0.31 | 0.77 | 0.03 | 0.01 | 0.02 |
| S-20 | 4 | 0.04 | 0.19 | 0.51 | 0.00 | 0.03 | 0.03 |
|  |  | 0.07±0.02 | 0.28±0.07 | 0.68±0.12 | 0.02±0.01 | 0.02±0.01 | 0.02±0.01 |

CAMHB VS CAMHB (4 mg/L cefoxitin): 2h (*P*=0.001), 3h (*P*=0.001), 4h (*P*=0.001).

Table S7. MSSA identification scores of different protocols in CHAMB (4 µg/mL cefoxitin).

| Number | MIC  (µg/mL) | Enrichment by non centrifugation | | | Enrichment by centrifugation | | |
| --- | --- | --- | --- | --- | --- | --- | --- |
|  |  | 2 h | 3 h | 4 h | 2 h | 3 h | 4 h |
| S-1 | 2 | - | - | - | - | - | - |
| S-2 | 4 | - | - | - | - | - | - |
| S-3 | 2 | - | - | - | - | - | - |
| S-4 | 2 | - | - | - | - | - | - |
| S-5 | 2 | - | - | - | - | - | - |
| S-6 | 2 | - | - | - | - | - | - |
| S-7 | 4 | - | - | - | - | - | - |
| S-8 | 4 | - | - | - | - | - | - |
| S-9 | 2 | - | - | - | - | - | - |
| S-10 | 4 | - | - | - | - | - | - |
| S-11 | 2 | - | - | - | - | - | - |
| S-12 | 4 | - | - | - | - | - | - |
| S-13 | 2 | - | - | - | - | - | - |
| S-14 | 4 | - | - | - | - | - | - |
| S-15 | 4 | - | - | - | - | - | - |
| S-16 | 4 | - | - | - | - | - | - |
| S-17 | 4 | - | - | - | - | - | - |
| S-18 | 2 | - | - | - | - | - | - |
| S-19 | 2 | - | - | - | - | - | - |
| S-20 | 4 | - | - | - | - | - | - |
|  |  | 0 | 0 | 0 | 0 | 0 | 0 |

The dash (-) indicates that the bacteria have not been conclusively identified.

A score >1.7 is considered valid in the species level and a score <1.7 indicates unreliable result.

Table S8. Concordance Rates of centrifuge enrichment combined with MALDI-TOF MS and microdilution broth by kappa test.

|  |  | microdilution broth method | |  |
| --- | --- | --- | --- | --- |
|  |  | MRSA | MSSA | Total |
| centrifuge enrichment combined with MALDI-TOF MS | MRSA | 20 | 0 | 20 |
|  | MSSA | 0 | 20 | 20 |
| Total | | 20 | 20 | 40 |

Table S9. The resistance of S. aureus to methicillin was identified by MALDI-TOF MS in the cerebrospinal fluid at 2h and 3h.

| Evaluation Index | Enrichment by non centrifugation | | Enrichment by centrifugation | |
| --- | --- | --- | --- | --- |
|  | 2 h | 4 h | 2 h | 4 h |
| Validity (%) | 0 | 92.5 | 70 | 100 |
| Sensitivity (%) | 0 | 85 | 40 | 100 |
| Specificity (%) | 0 | 100 | 100 | 100 |
| Positive predictive value (%) | 0 | 100 | 100 | 100 |
| Negative predictive value (%) | 0 | 86.9 | 80 | 100 |

Table S10. Sources and WGS data of the 40 clinical *S. aureus* isolates.

| Strain | Source​ | WGS | Strain | Source​ | WGS |
| --- | --- | --- | --- | --- | --- |
| R-1 | CSF | MRSA | S-1 | Secretion | MSSA |
| R-2 | CSF | MRSA | S-2 | CSF | MSSA |
| R-3 | CSF | MRSA | S-3 | CSF | MSSA |
| R-4 | Sputum | MRSA | S-4 | CSF | MSSA |
| R-5 | CSF | MRSA | S-5 | Sputum | MSSA |
| R-6 | CSF | MRSA | S-6 | Secretion | MSSA |
| R-7 | Secretion | MRSA | S-7 | CSF | MSSA |
| R-8 | Sputum | MRSA | S-8 | Secretion | MSSA |
| R-9 | CSF | MRSA | S-9 | CSF | MSSA |
| R-10 | CSF | MRSA | S-10 | CSF | MSSA |
| R-11 | Secretion | MRSA | S-11 | CSF | MSSA |
| R-12 | CSF | MRSA | S-12 | Sputum | MSSA |
| R-13 | Sputum | MRSA | S-13 | Secretion | MSSA |
| R-14 | CSF | MRSA | S-14 | CSF | MSSA |
| R-15 | CSF | MRSA | S-15 | CSF | MSSA |
| R-16 | Secretion | MRSA | S-16 | CSF | MSSA |
| R-17 | CSF | MRSA | S-17 | Sputum | MSSA |
| R-18 | CSF | MRSA | S-18 | CSF | MSSA |
| R-19 | Sputum | MRSA | S-19 | CSF | MSSA |
| R-20 | CSF | MRSA | S-20 | CSF | MSSA |

WGS, Whole Genome Sequencing

Table S11. Limit of detection for *S. aureus* identification and methicillin resistance differentiation^a^.

| OD_600_ values | Enrichment by non centrifugation | | | | Enrichment by centrifugation | | | |
| --- | --- | --- | --- | --- | --- | --- | --- | --- |
|  | No. of spots identified^b^ | | | % | No. of spots identified | | | % |
| 0.40  0.20  0.10  0.05  0.02 | 5  3  1  0  0 | 5  4  1  0  0 | 5  3  2  0  0 | 100  66.7  26.7  0  0 | 5  5  4  2  0 | 5  5  4  1  0 | 5  5  5  1  0 | 100  100  86.7  26.7  0 |

^a^Each sample was analyzed three times, each time adding 5 parallel spots on the target plate.

^b^Out of 5 target spots.
